# Frenemies: Interactions between Rhizospheric Bacteria and Fungi from Metalliferous Soils

**DOI:** 10.3390/life11040273

**Published:** 2021-03-25

**Authors:** Stefano Rosatto, Grazia Cecchi, Enrica Roccotiello, Simone Di Piazza, Andrea Di Cesare, Mauro Giorgio Mariotti, Luigi Vezzulli, Mirca Zotti

**Affiliations:** 1 Laboratory of Plant Biology, DISTAV-Department of Earth, Environment and Life Sciences (DISTAV), University of Genoa, Corso Europa 26, 16132 Genova, Italy; stefano.rosatto@unige.it (S.R.); enrica.roccotiello@unige.it (E.R.); m.mariotti@unige.it (M.G.M.); 2 Laboratory of Mycology, DISTAV-Department of Earth, Environment and Life Sciences (DISTAV), University of Genoa, Corso Europa 26, 16132 Genova, Italy; simone.dipiazza@unige.it (S.D.P.); mirca.zotti@unige.it (M.Z.); 3 MEG—Molecular Ecology Group, National Research Council of Italy (CNR-IRSA), Water Research Institute, Largo Tonolli 50, 28922 Verbania, Italy; andrea.dicesare@cnr.it; 4 Laboratory of Microbiology, DISTAV-Department of Earth, Environment and Life Sciences (DISTAV), University of Genoa, Corso Europa 26, 16132 Genova, Italy; luigi.vezzulli@unige.it

**Keywords:** bacteria-fungal interactions, biofilm, co-growth, *Pseudomonas fluorescens*, *Streptomyces vinaceus*, *Penicillium ochrochloron*, *Trichoderma harzianum* group, rhizosphere

## Abstract

Is it possible to improve the efficiency of bioremediation technologies? The use of mixed cultures of bacteria and fungi inoculated at the rhizosphere level could promote the growth of the associated hyperaccumulating plant species and increase the absorption of metals in polluted soils, broadening new horizons on bioremediation purposes. This work investigates interactions between Ni-tolerant plant growth-promoting bacteria and fungi (BF) isolated from the rhizosphere of a hyperaccumulating plant. The aim is to select microbial consortia with synergistic activity to be used in integrated bioremediation protocols. *Pseudomonas fluorescens* (*Pf*), *Streptomyces vinaceus* (*Sv*) *Penicillium ochrochloron* (*Po*), and *Trichoderma harzianum* group (*Th*) were tested in mixes (*Po-Sv*, *Po-Pf*, *Th-Pf*, and *Th-Sv*). These strains were submitted to tests (agar overlay, agar plug, and distance growth co-growth tests), tailored for this aim, on Czapek yeast agar (CYA) and tryptic soy agar (TSA) media and incubated at 26 ± 1 °C for 10 days. BF growth, shape of colonies, area covered on plate, and inhibition capacity were evaluated. Most BF strains still exhibit their typical characters and the colonies separately persisted without inhibition (as *Po-Sv*) or with reciprocal confinement (as *Th-Sv* and *Th-Pf*). Even if apparently inhibited, the *Po-Pf* mix really merged, thus obtaining morphological traits representing a synergic co-growth, where both strains reached together the maturation phase and developed a sort of mixed biofilm. Indeed, bacterial colonies surround the mature fungal structures adhering to them without any growth inhibition. First data from in vivo experimentation with *Po* and *Pf* inocula in pot with metalliferous soils and hyperaccumulator plants showed their beneficial effect on plant growth. However, there is a lack of information regarding the effective co-growth between bacteria and fungi. Indeed, several studies, which directly apply the co-inoculum, do not consider suitable microorganisms consortia. Synergic rhizosphere BFs open new scenarios for plant growth promotion and soil bioremediation.

## 1. Introduction

Bacteria and fungi are simultaneously present in a wide variety of environments [1]. These organisms are capable not only of coexisting, but also of actively interacting and the bacteria-fungal interactions (BFIs) range from antagonism to mutualism [2,3]. BFIs in many cases form physically and metabolically interdependent consortia with peculiar characteristics different from those of their individual components [3].

Bacteria and fungi affect their microenvironment by producing secretes and metabolites to compete with each and/or other organisms [2,3]. However, they can also live in synergistic communities, cooperating to survive and thrive together [1,4,5,6].

BFIs play a key role in most of natural ecosystems, driving biogeochemical cycles, contributing to both the health and diseases of plants and animals [2], as well as degrading and inactivating pollutants [7].

Beneficial effects of soil biotic components are often applied in the eco-friendly bioremediation techniques that use living organisms (i.e., bacteria, fungi, and plants) to remove or neutralize hazardous organic and/or inorganic compounds in polluted sites [8,9,10]. These technologies can exploit native organisms to detoxify and remove pollutants from soil, through their different metabolic capabilities: enzymes, organic acids, and secondary metabolites [11,12]. Recently, many studies highlighted the beneficial effects of inoculating microbial consortia at the root level to reduce toxic effects and concentration of persistent organic and inorganic contaminants (e.g., metals) [13,14,15,16,17,18].

Rhizospheric plant growth-promoting bacteria (PGPB) and plant growth promoting fungi (PGPF) are used because of their interactions with roots (e.g., increasing solubility of nutrient trace elements, root surface area and root hair development, accumulation of metals) of different plants species [19,20,21,22,23,24].

Soil microorganisms may biotransform metals into less toxic forms uptaken by plants and they are also able to release plant growth regulators [25], such as 1-aminocyclopropane-1-carboxylic Acid (ACC) deaminase, indole-3-acetic acid (IAA), synthesize siderophores and solubilize and mineralize phosphate [26,27,28,29].

It is known that plant-associated microbiota play an important role in plant trace elements bioaccumulation [30,31]. BFIs, in fact, are part of a communication network that keeps microhabitats in balance [32]. The synergic activity of certain fungi and bacteria has been already documented, demonstrating the improved ability of BFIs in remediation of hydrocarbons and crude oil [33,34,35,36].

Despite the great potential of plant growth-promoting (PGP) microorganisms, little is still known about their mutual behavior and possible positive interactions between fungi and PGP bacteria. To date, studies have focused mainly on the evaluation of the antagonistic properties of bacterial isolates against fungal isolates whilst possible synergic effect of bacteria-fungi interactions has generally been neglected [37,38].

Recently, we isolated and characterized bacterial and fungal strains in metalliferous soils [24] from the rhizosphere of *Alyssoides utriculata* (L.) Medik., a nickel hyperaccumulator plant [39]. Among the isolated strains, we selected the most performing culturable bacteria and fungi in terms of Ni tolerance and PGP traits: *Pseudomonas fluorescens* Migula, 1895 complex, *Streptomyces vinaceus* Jones, 1952, *Penicillium ochrochloron* Biourge 1923, and *Trichoderma harzianum* Rifai 1969 group strains that were the most promising for integrated nickel bioremediation [24,40,41,42].

This work investigates the possible mutual interactions between the above mentioned bacterial (*P. fluorescences*, *S. vinaceus*) and fungal strains (*P. ochrochloron* and *T. harzianum* group) in vitro and assesses in vivo effect in Ni-hyperaccumulator plants on metalliferous soils with the aim of selecting synergic microbial consortia employable for integrated plant-fungi-bacteria bioremediation purposes.

## 2. Materials and Methods

### 2.1. Organisms Selection and In Vitro Co-Growth Tests

The used strains were deposited in the microbial Collection of DISTAV of the University of Genoa, associated member of the Microbial Resource Research Infrastructure Italian Node (CoLD-MIRRI-IT-UNIGE), and were previously isolated and characterized for the high Ni tolerance and the PGP traits [24]. The high Ni tolerance of *Pseudomonas fluorescens* SERP1 complex, *Streptomyces vinaceus* SERP4, *Penicillium ochrochloron* Serp03S, and *Trichoderma harzianum* group Serp05S was evaluated on Tryptic Soy Agar (TSA, Sigma-Aldrich, St. Louis, MO, USA) spiked with Ni up to 20 mM [24]. Thanks to these traits, strains were used in the following co-growth tests.

Bacterial strains were grown in cell culture flasks containing tryptic soy broth (TSB, Sigma Aldrich) until reaching the exponential growth phase. The first strain was incubated overnight at 26 ± 1 °C, while the second was kept at 26 ± 1 °C for 72 h, then revived 1:20 in the same growth medium and finally incubated for 30 h. Bacterial cells pellets were washed twice in 1× phosphate-buffered saline (PBS) solution pH 7.4 and then concentrated [43] to obtain the final concentration of 10^8^ Colonies Forming Units (CFUs) mL^−1^ measured spectrophotometrically.

Fungal strains of *P. ochrochloron* and *T. harzianum* group were plated on malt extract agar (MEA) and kept at 24 ± 1 °C until conidiogenesis. In addition to the pure fungal culture on MEA agar, an aliquot of well-grown fungal colony was diluted in distilled sterile water and estimated by cell counts using a Bürker chamber up to a liquid culture concentration of 10^8^ CFUs mL^−1^.

For each bacterium and each fungus previously selected, in addition to the controls, three methods of inoculum were adopted onto two different substrates Czapek Yeast extract Agar (CYA) and TSA at pH 6, and each method was carried out in triplicate (N = 30 each mixed culture test): (a) agar overlay method (100 µL of fungal liquid culture were spread on solid medium and 100 μL of bacterial suspension were inoculated at the center of the Petri dishes); (b) agar plug method (Ø 5 mm agar disc from well-grown lawn of the fungus was cut and transferred on agar plate previously stroked with 100 μL of the bacterial suspension); (c) distance growth method (Ø 5 mm fungal agar plug and 100 µL of the bacterial suspension were inoculated 3 cm away from each other on the same plate). Agar plates (90 mm Ø) with a single central culture of the selected bacteria and fungi were used as positive control.

*P. fluorescens* SERP1 complex and *S. vinaceus* SERP4 were grown in cell culture flasks containing tryptic soy broth (TSB, Sigma-Aldrich) as previously described to reach the exponential growth phase.

*P. ochrochloron* Serp03S and *T. harzianum* Serp05S group were plated on MEA agar and kept at 24 ± 1 °C until conidiogenesis. The different bacterium-fungus mixes and the positive controls were then incubated at 26 ± 1 °C and the growth of the isolates was daily monitored for 10 days.

Mycelial and bacterial growth was assessed by measuring colony area and the percentage inhibition (*Pi*%) [44], calculated according to the formula:

(1) Pi%=[(C−T)× 100]/C

where *Pi*% = percentage inhibition ratio, *C* = average control area (mm^2^) of the colony and *T* = average treatment area (mm^2^) of the colony.

### 2.2. Stereomicroscopy and Optical Microscopy

The Petri dishes were observed under a stereomicroscope and the images were processed with the Leitz(sche) Camera (LEICA, Wetzlar, Germany) application suite (LAS EZ) software to monitor the co-growth of the microbiota after 10 days of incubation and the corresponding shape of both colonies. Subsequently, the in-depth observation of microfungal structures of culturable mix was appreciated through an optical microscope magnified at 40× and 63× and the resulting images were processed using the LEICA IM50 software, while the Gram staining allowed us to underline the Gram+ and Gram- bacteria.

### 2.3. Scanning Electron Microscopy

In order to carefully observe the best grown mixed culture of microbiota after 14 days-incubation on CYA, the isolates mix were fixed in phosphate 10% buffered formalin and dehydrated in ethanol series [45]. The samples were then mounted on SEM stubs, sputter-coated with gold and viewed with a VEGA3 SEM (TESCAN, Libušina, Czech Republic) at HV20.0 kV, using backscattered electrons (BSE).

### 2.4. Soil Sampling and Processing for In Vivo Experimentation

Serpentine soil was collected from Sassello (SV, Italy N 44°48′63″; E 8°51′87″) at 0–30 cm (A horizon) and 30–60 cm (C horizon) depth, mixed to obtain a homogeneous sample, air de-hydrated and sieved through a sequential 4 mm and 2 mm mesh. One soil fraction was used as is for Control, the other fractions were oven dried at 130° for 24 h to sterilize the growing substrate, then mixed 1:1 (*v*/*v*) to obtain the testing substrates for pot experiment. Soil chemical composition is reported [46], as shown following in Table 1.

### 2.5. Plant Preparation for In Vivo Experimentation

Seeds of Ni-hyperaccumulator *Alyssoides utriculata* (n = 200) were collected from a serpentine area in NW Italy (Voltri Massif N 44°28′49″, E 8°40′44″) and grown in greenhouse on sterilized serpentine soil (Section 2.4) for 6 months.

### 2.6. In Vivo Experimental Design

Four L of substrate prepared as previously described (approx. 4.5 kg each pot) was divided into Control, Bacteria (BI, inoculum with *Pseudomonas fluorescens*), Fungi (FI inoculum with *Penicillium ochrocloron*) and Mix (MIX co-inoculum *Po* + *Pf*), 30 replicates each series. Soils were hydrated at 70% water holding capacity and subsequently irrigated once a week to keep them at optimum hydration level. After three months from the inocula, pots series were completely randomized, and six-months old plants were then transplanted in the pots. All the pots had their saucers to recover the irrigation water.

### 2.7. Bacteria and Fungi Co-Inoculum in In Vivo Experimentation

The selected *P. ochrochloron* strain (FI) was isolated as pure colony on MEA (Sigma-Aldrich) and incubated in the dark at 24 ± 1 °C for at least one week. Once the pure colony has been grown and the conidiogenesis was reached, mycelium and conidia were collected with sterile loop and resuspended in 100 mL of sterile distilled water up to a final concentration of about 10^8^ CFUs mL^−1^. To verify the final fungal concentration, aliquots of this latter solution were transferred to the Bürker chamber for conidia count.

*P. fluorescens* strain (BI) was repeatedly isolated to obtain a pure colony on TSA (Sigma-Aldrich) incubated at 26 ± 1 °C in the dark for at least 24–48 h. One day before the experiment, a well isolated colony is transferred to a cell culture flask containing 200 mL of TSB (Sigma-Aldrich) and incubated overnight at 26 ± 1 °C. The subsequent day, the cultural broth is transferred into 50 mL falcon tubes which are centrifuged at 4500× *g* for 10 min at 20 °C. Then, the supernatant is gently removed from each falcon tube and the pellet is subjected to washing with an equal volume of PBS (pH 7.4). A spectrophotometer analysis allows to determine the optical density (OD) at a wavelength of 600 nm. Finally, the pellet obtained after a second centrifugation is resuspended in 100 mL of physiological saline solution. After an overnight incubation of *P. fluorescens* at 26 ± 1 °C the obtained OD is 0.5, which corresponds to an average concentration of 2.5 × 10^8^ CFUs mL^−1^ of the Gram-negative bacterium *P. fluorescens*. Furthermore, the bacterial suspension is concentrated twice, to obtain a final concentration of 5 × 10^8^ CFUs mL^−1^.

The BI and FI inoculation in pots is carried out immediately after their preparation, transferring 1 mL of bacterial and fungal suspension of about 10^8^ CFUs mL^−1^ on the soils in the pot centre.

### 2.8. Microbiota Assessment

The fungal and bacterial diversity and abundance were monitored at month I (without plants) and XII (with six-months old plants) after the inocula. Soil was sampled in each pot and mixed for each treatment to obtain a composite sample. Soils samples were collected for every type of treatment: fungal inoculum (FI), bacteria inoculum (BI), fungal-bacteria co-inoculum (MIX), native soil without inocula (Control). Microorganisms were extracted from 3 g of soil by using 30 mL of sterile saline solution (NaCl 0.9% w/v). Aliquots were serially diluted (1:10) up to 10^−5^ and 100 µL of each dilution was spread on Rose Bengal Agar (RBA) and MEA. Petri dishes (Ø 90 mm) for fungi and TSA (Sigma-Aldrich) for bacteria were employed. Then, fungal plates (N = 24) were incubated in the dark at 24 ± 1 °C and monitored every day for a week, while bacterial plates (N = 16) were incubated in the dark at 26 ± 1 °C for 72–96 h. The fungal and bacterial colonies were counted as Colonies Forming Units per g of dry soil (CFUs g^−1^) for each morphotype grown and repeatedly isolated on agar plates to obtain a pure colony.

### 2.9. Data Analysis

For each treatment, the images of the bacterial and fungal colonies were processed with ImageJ software [47,48] to estimate the surface area. Data are presented as mean ± Standard Deviation. The percentage of the inhibition rate was obtained considering the average surface of the bacterial and fungal colonies.

## 3. Results

### 3.1. In Vitro Tests

The screening tests of co-growth showed that CYA and TSA buffered at pH 6.0 resulted the most suitable agar substrates for the growth of both bacterial and fungal strains.

Fungal strains exhibited an increased growth on the CYA substrate compared to TSA pH 6.0, whereas bacteria thrive on both agar culture media.

**Figure 1:**
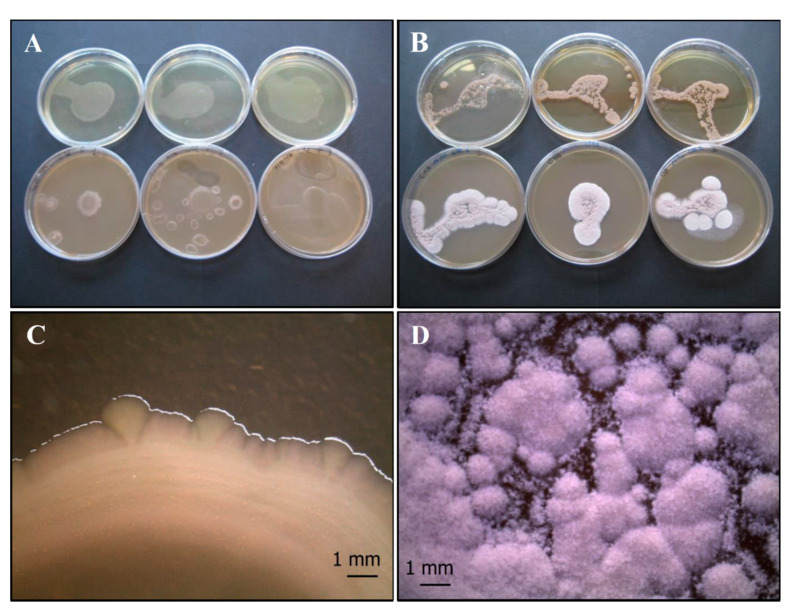
Growth of control bacterial strains *P. fluorescens* complex SERP1 (**A**,**C**) and *S. vinaceus* SERP4 (**B**,**D**) on TSA (upper row of plates, **A** and **B**, respectively) and on CYA (lower row of plates, **A** and **B**, respectively). Stereo Microscope details of single colony shape of *P. fluorescens* (**C**) that forms small, low convex, colorless or white-cream colonies, and *S. vinaceus* (**D**) that forms circular, convex, and rough colonies with a macroscopic mycelium morphology including pellets and clump formation. N = 30 each test.

*P. fluorescens* (*Pf*) forms small, low convex, colorless colonies, although white-cream pigmentation is frequent [49] (Figure 1A,C), while *S. vinaceus* (*Sv*) forms slow-growing, circular, convex, and rough colonies (Figure 1B,D). This latter bacterium is characterized by a macroscopic mycelium morphology including pellets and clump formation [50].

**Figure 2:**
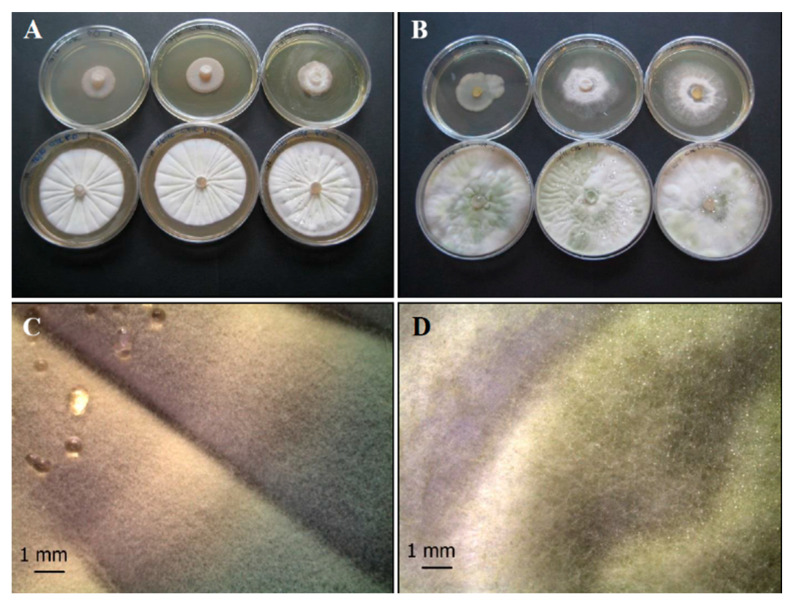
Growth of control fungal strains *P. ochrochloron* Serp03S (**A**,**C**) and *T. harzianum* group Serp05S (**B**,**D**) on TSA (upper row of plates, **A** and **B**, respectively) and on CYA (lower row of plates, **A** and **B**, respectively). Stereo Microscope details of single colony shape of *P. ochrochloron* (**C**) that appears round, grooved in a radial pattern, white to light grey in the margin once maturation is reached and *T. harzianum* (**D**) that produces typical rough and globose to subglobose yellow or green conidia. N = 30 each test.

As concerns fungi, the colony of *P. ochrochloron* (*Po*) on CYA appears round, grooved in a radial pattern, white to light grey in the margin once maturation is reached (Figure 2A,C). This fungus exhibits fast growth rate, and its colonies are variously branched, with asymmetrically and/or symmetrically branched conidiophores [51].

*T. harzianum* group (*Th*) isolates (Figure 2B,D) produce typical rough and globose to subglobose conidia and their color was yellow or green [52,53,54]; conidia production is greater in the middle of the plate with respect to the margins.

The results of co-growth tests are summarized as follows:

(a) *P. ochrochloron* (Serp03S) vs. *P. fluorescens* (SERP1): *Po-Pf* mix

**Figure 3:**
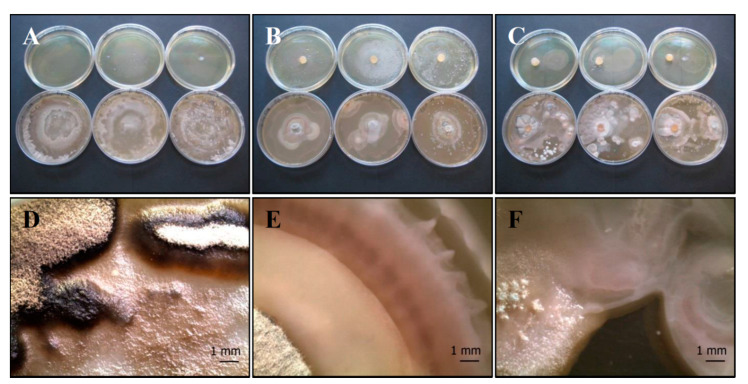
Growth of *P. ochrochloron* Serp03S and *P. fluorescens* complex SERP1 (*Po-Pf* mix) in co-growth tests on TSA (upper row of plates, **A**–**C**, respectively) and on CYA (lower row of plates, **A**–**C**, respectively). Method of inoculum: (**A**) agar overlay. (**B**) agar plug, (**C**) distance growth. Stereo Microscope details of colony shape when grown in mix (**D**–**F**). In the agar overlay method, *Po* grows around *Pf* reaching maturation and its color turns to grey-black (**D**). In the agar plug method (**E**) and in the distance growth method (**F**) the contact between the two organisms develops a peculiar round structure with concentric rings. N = 30 each test.

The agar overlay method (Figure 3A,D) on CYA does not seem to clarify any relationship between the two organisms. The bacterium develops where it has been inoculated, while the fungus grows better around bacterium. After 10 days of incubation the fungus reaches maturation, taking on a grey-black color.

Interestingly, in the agar plug (Figure 3B,E) and in the distance growth methods (Figure 3C,F) the contact between the two organisms develops a peculiar round structure with concentric rings that represents a sort of synergic growth.

(b) *P. ochrochloron* (Serp03S) vs. *S. vinaceus* (SERP4): *Po-Sv* mix

**Figure 4:**
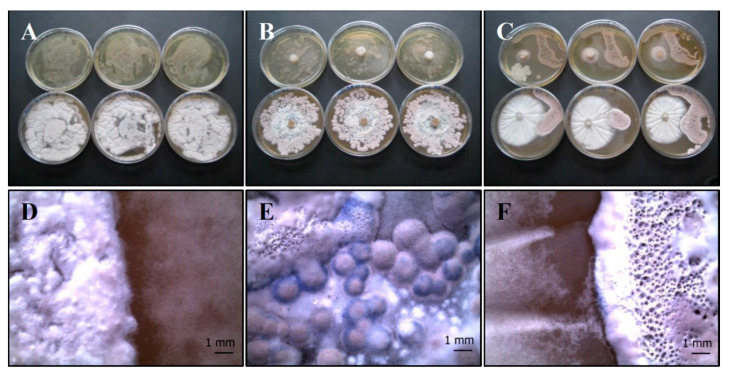
Growth of *P. ochrochloron* Serp03S and *S. vinaceus* complex SERP4 (*Po-Sv* mix) in co-growth tests on TSA (upper row of plates, **A**–**C**, respectively) and on CYA (lower row of plates, **A**–**C**, respectively). Method of inoculum: (**A**) agar overlay, (**B**) agar plug, (**C**) distance growth. Stereo Microscope details of colony shape when grown in mix (**D**–**F**). In the agar overlay and, in the agar plug method the mixed culture turns on grey-white color (**A**,**B**). In the distance growth method (**F**) no zones of inhibition are visible, specifically in the contact zone, but each strain maintained a distinct colony shape in terms of macroscopic characteristics. N = 30 each test.

The mixed culture covers almost the entire agar plate, taking on a grey-white color both in the overlay (Figure 4A,D) and plug test (Figure 4B,E). No zones of inhibition are visible in the distance growth method, specifically in the contact zone (Figure 4C,F), but each strain maintained a distinct colony shape in terms of macroscopic characteristics.

(c) *T. harzianum* group (Serp05S) vs. *P. fluorescens* complex (SERP1): *Th-Pf* mix

**Figure 5:**
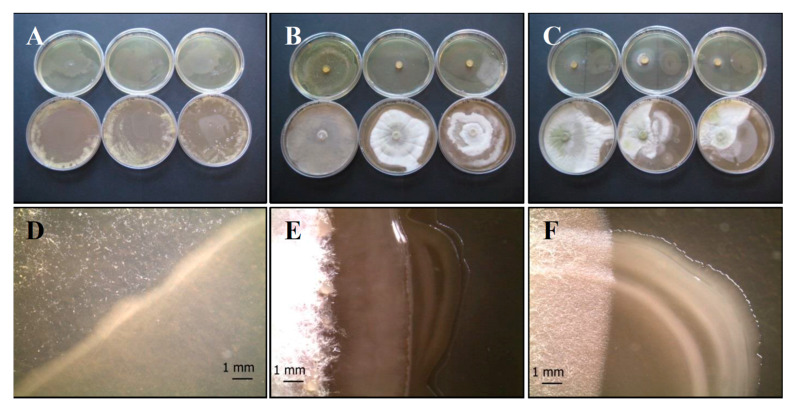
Growth of *T. harzianum* Serp05S and *P. fluorescens* complex SERP1 (*Th-Pf* mix) in co-growth tests on TSA (upper row of plates, **A**–**C**, respectively) and on CYA (lower row of plates, **A**–**C**, respectively). Method of inoculum: (**A**) agar overlay, (**B**) agar plug, (**C**) distance growth. Stereo Microscope details of colony shape when grown in mix (**D**–**F**). In the agar overlay method the fungus does not thrive where the bacterium was inoculated (**D**). In the distance growth method, *Th* growth is disturbed and confined in its half of the plate (**C**,**F**). N = 30 each test.

In the agar overlay method the fungus does not thrive where the bacterium was inoculated (Figure 5A,D). Conversely in the other treatments (agar plug, Figure 5B,E and distance growth, Figure 5C,F) the fungus covers almost the entire agar plate, but its growth appears to be disturbed: in the last distance growth method, fungal strain is confined in its half of the plate.

(d) *T. harzianum* (Serp05S) vs. *S. vinaceus* (SERP4): *Th-Sv* mix

**Figure 6:**
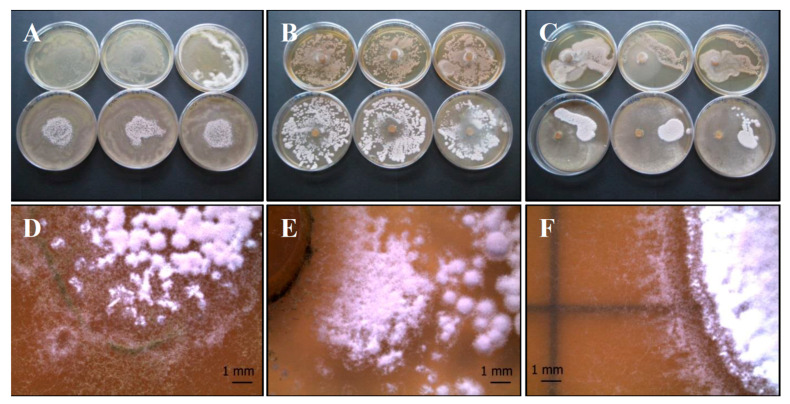
Growth of *T. harzianum* Serp05S and *S. vinaceus* complex SERP4 (*Th-Sv* mix) in co-growth tests on TSA (upper row of plates, **A**–**C**, respectively) and on CYA (lower row of plates, **A**–**C**, respectively). Method of inoculum: (**A**) agar overlay, (**B**) agar plug, (**C**) distance growth. Stereo Microscope details of colony shape when grown in mix (**D**–**F**). In the agar overlay method, *Sv* only grows in the center of the plate (**D**). In the agar plug method, *Th* appears to be inhibited (**E**). In the distance growth method, *Th* and *Sv* tolerates each other without reciprocal inhibition in the contact zone but persists distinct (**F**). N = 30 each test.

The bacterial colonies appear smaller than the control and the fungus reaches maturation later than pure colonies. The bacterium grows only in the center of the plate in the overlay agar test (Figure 6A,D), while the fungus appears to be inhibited in the agar plug test (Figure 6B,E). Bacteria and fungi tolerate each other, as shown by the contact between the two microorganisms (Figure 6C,F), but they remain distinct.

#### 3.1.1. Stereomicroscopy and Optical Microscopy

**Figure 7:**
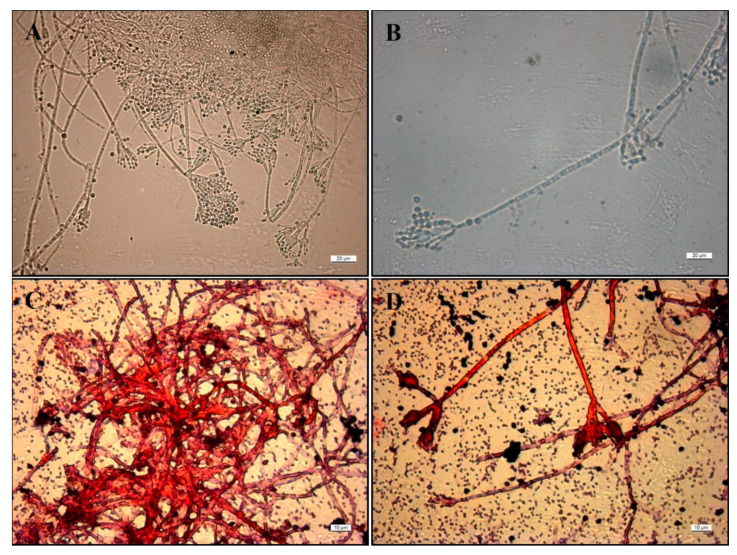
Optical Microscope images (40×) of (**A**) mono- and biverticillate branched conidiophores of *P. ochrochloron* Serp03S in the co-growth method and (**B**) maturation of conidia on conidiophores, in detail. Gram staining images of *P. ochrochloron* Serp03S and *P. fluorescens* SERP1 by Optical Microscope at 63× magnification. In detail (**C**) the mass of fungal hyphae and (**D**) the branched conidiophores of *P. ochrochloron* surrounded by bacterial cells that do not retain the crystal violet stain used in the Gram-staining method (pink-red rods).

In-depth observation of the best co-grown bacterium and fungus mix (*Po-Pf* mix) under the optical microscope (Figure 7A,B) shows the typical fungal conidiophore branching patterns mostly symmetrical and mono- and biverticillated [55]. The Gram staining underlines the Gram-negative bacterium *P. fluorescens* complex, counter-stained pink by safranin (Figure 7C,D). However, the conidiophores of *P. ochrochloron* are also clearly stained with a bright red color.

#### 3.1.2. Scanning Electron Microscopy

**Figure 8:**
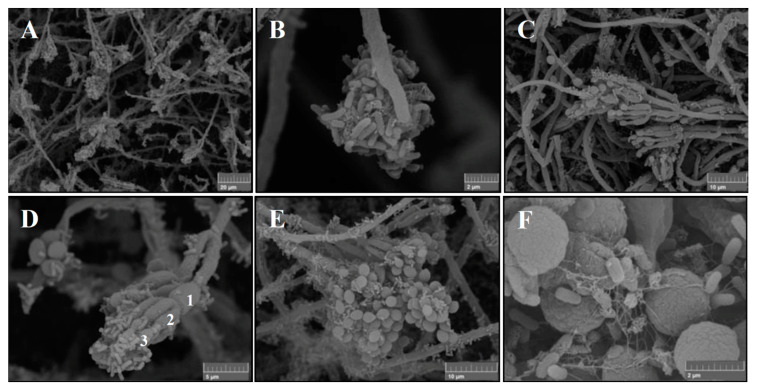
Backscattered electron (BSE) SEM images of the mixed culture of *P. ochrochloron* Serp03S and *P. fluorescens* SERP1: (**A**) tangle of fungal hyphae covered by bacteria. Magnification 1.90 kx, Working Distance 10.51 mm; (**B**) growing hyphal tip surrounded by cluster of bacteria jointed by means of adhesins. Magnification 19.8 kx, Working Distance10.84 mm; (**C**) large group of branching biverticillated conidiophores. Magnification 4.63 kx, Working Distance 10.66 mm; (**D**) detail of branch (1) metula (2) interfaces with bacteria adherent to phialides (3). Magnification 10.6 kx, Working Distance 10.51 mm; (**E**) mature conidia on conidiophores. Magnification 20.0 kw, Working Distance 10.57 mm; (**F**) fungal conidia (2 µm diameter) and bacterial colonies (1 µm length), some of which in death phase; the rough surface of the conidia seems to encourage the adhesion of the bacteria cells.

The backscattered (BSE) SEM imaging of the best co-grown bacterium and fungus mix (*Po-Pf* mix) highlights clusters of fungal hyphae enveloped by groups of flagellate *Pf* (Figure 8A,B). Conidiophores are often covered on the tip by bacterial colonies adhering by means of adhesive filaments that provide a compact structure to bacterial biofilm consisting of live and dead cells (Figure 8C,D). Fungal spherical conidia are still borne (Figure 8E) or free (Figure 8F) on specialized stalks of the conidiophore that is characterized by typical vial-shaped cell (i.e., phialide), as shown in Figure 8D.

#### 3.1.3. Mutual Growth Inhibition

The analysis of bacterial and fungal growth reveals that the measured area is a very critical parameter to evaluate the inhibition of the colonies’ growth (Table 2).

Fungi grow slower than bacteria, but the expansion over time of mycelium is greater than that of bacterial colonies. Therefore, fungal strains show a greater surface coverage than the bacterial one. *T. harzianum* group covers the entire round surface area of the agar plate (90 mm Ø) and *P. ochrochloron* occupies about 70% of the surface area of the circle.

Conversely, the bacterial strains show a limited growth close to the initial bacterial suspension (~25% of the plate surface). As shown in Table 3, *P. fluorescens* shows a marked reduction in colony area in the co-growth treatment with both *P. ochrochloron* (>50% for the average area) and *T. harzianum* group (>70% for the average area).

However, this result is difficult to explain in the distance growth test with *P. ochrochloron*: the development of a sort of synergic growth between these two microorganisms does not allow to distinguish the distinct shape of the two colonies that completely merged. This phenomenon was also observed by optical and scanning electron microscopy. On the contrary, *S. vinaceus* is less sensitive to co-growth with other fungal strains, showing a low percentage of inhibition. This is especially true for the test between *S. vinaceus* and *P. ochrochloron* (~8% inhibition). The fungus instead shows a surface area variation roughly ranging from 30 to 40%. Similar findings for all the treatments including *T. harzianum* group, which shows signs of suffering with respect to the control.

### 3.2. In Vivo Experimentation

#### 3.2.1. Assessment of Culturable Fungi

**Figure 9:**
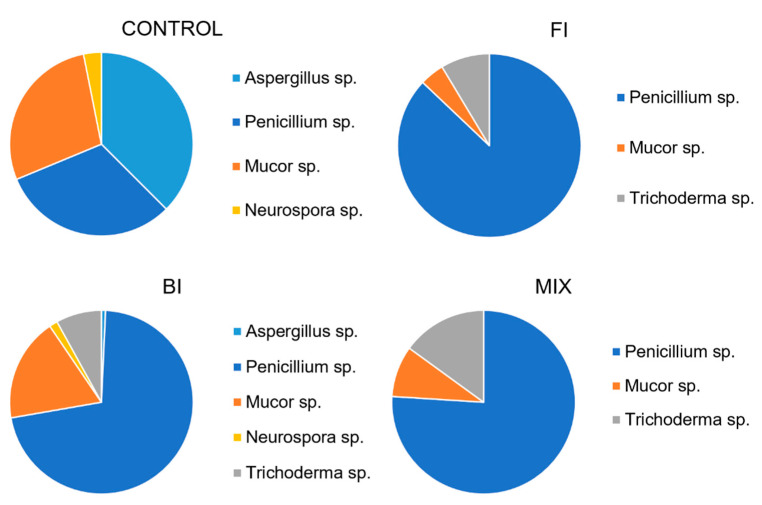
Average fungal count expressed as genus diversity and colony forming units (CFUs g^−1^) in the rhizospheric soil one-month post inoculation: control, fungal inoculum; bacterial inoculum; MIX treatment. N = 16 each treatment.

One month from the inoculation five different fungal morphotypes were recognized in the plated soils. The fungal abundance was very high in the control and in the BI samples. The main representative fungus morphotype was *Penicillium* cfr. *ochrochloron*. Other fungi were *Trichoderma* strain, isolated in each soil sample except the control; *Neurospora* sp., isolated in each sample except the FI and MIX; *Mucor* sp. isolated in each sample, and *Aspergillus* sp. isolated in control and BI samples. The abundance of each morphotype is reported in Figure 9.

**Figure 10:**
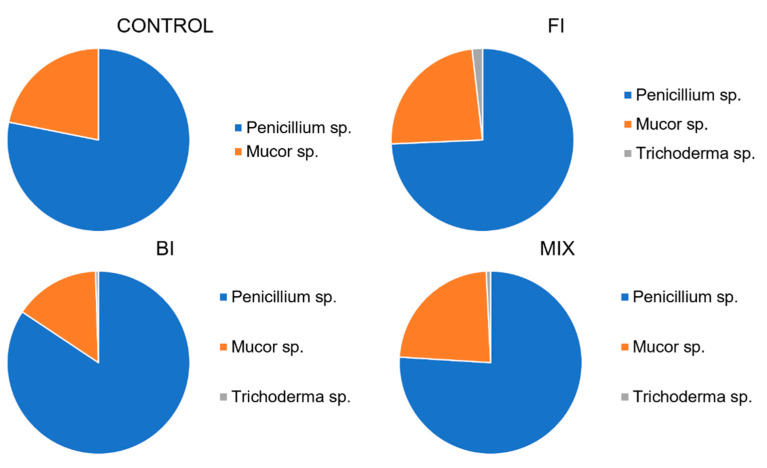
Average fungal count expressed as genus diversity and colony forming units (CFUs g^−1^) in the rhizospheric soil twelve-months post inoculation: control, fungal inoculum; bacterial inoculum; MIX treatment. N = 16 each treatment.

Twelve months after the inoculation, three different fungal morphotypes were identified in the plates. The fungal abundance was homogenized in the samples, but the *Penicillium* cfr. *ochrochloron* was the most represented species, followed by *Mucor* sp. and *Trichoderma* sp. No *Aspergillus* sp. and *Neurospora* sp. strains were isolated from the plates. The abundance of each morphotype is reported in Figure 10.

#### 3.2.2. Assessment of Culturable Bacteria

**Figure 11:**
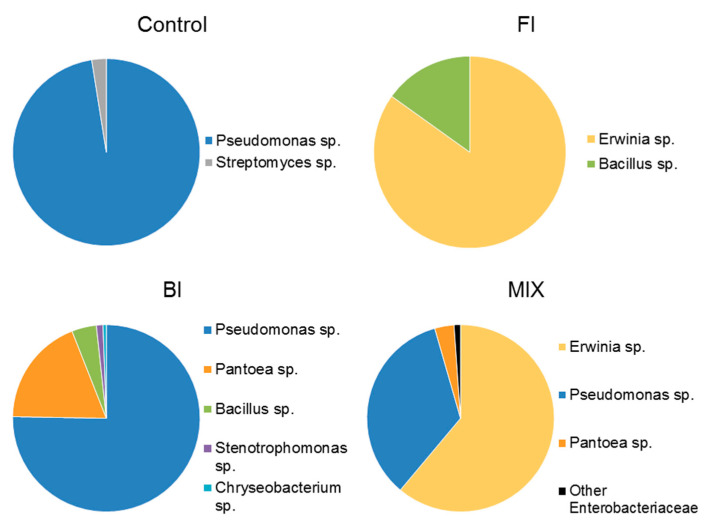
Average bacterial count expressed as genus diversity and colony forming units (CFUs g^−1^) in the rhizospheric soil one-month post inoculation: control, fungal inoculum; bacterial inoculum; MIX treatment. N = 16 each treatment.

One month after inoculation a greater diversity is observed, where *P. fluorescens* has been added to the soil (BI and MIX) compared to control and FI treatments (Figure 11). In general, there is a prevalence of genus *Pseudomonas* sp. which represents more than 75% of total culturable bacterial strains in BI, while it stands around 34% in the MIX.

It is interesting to note that the dominant bacterial strain in FI (~85%) is *Erwinia* sp., while the Pseudomonadaceae are dominant in control (97.5%).

**Figure 12:**
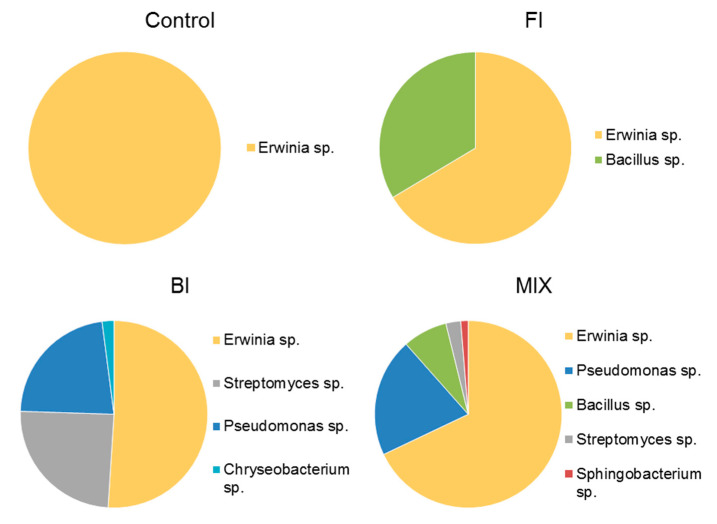
Average bacterial count expressed as genus diversity and colony forming units (CFUs g^−1^) in the rhizospheric soil twelve-months post inoculation: control, fungal inoculum; bacterial inoculum; MIX treatment. N = 16 each treatment.

After twelve months, although there are no significant differences on the isolated morphotypes, *Pseudomonas* sp. it is no longer the most abundant genus in BI (−22%). In fact, *Streptomyces* sp. (−24%) and *Erwinia* sp. thrive in all types of treated soils. In fact, the pie chart in Figure 12 shows that *Erwinia* sp. represents half of the bacterial colonies isolated in BI, 66% and 68% in FI and MIX respectively and even represents the monospecific community in control.

#### 3.2.3. Plant Growing

**Figure 13:**
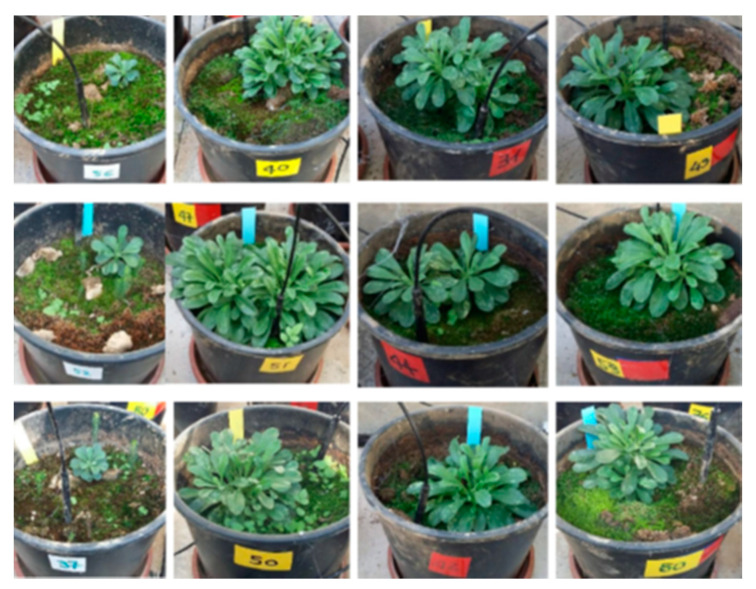
Plants of *Alyssoides utriculata* six months after transplanting in control soil, and in soil inoculated with bacteria (BI), fungi (FI) and MIX (Pf + Po).

Six months after transplanting, plants from inoculated or co-inoculated pots are markedly different respect to control. These latter showed reduced growth with absence of secondary branches and were about 10 times smaller than inoculated plants (Figure 13).

The overall growth was MIX > BI > FI > CONTROL.

## 4. Discussion

The co-growth tests between bacterial and fungal strains allow establishing the positive or negative interactions and the mutual behavior of the selected strains. Most of the bacterial and fungal strains used in mixes showed typical macro- and micromorphology where the two strains separately persisted without inhibition (as in *Po*-*Sv*), or with reciprocal confinement sometimes developing slower than expected (as in *Th-Sv* and *Th-Pf*). On the contrary, this study highlighted for the first time the morphological traits that represent a sort of synergic co-growth, as in the *Po*-*Pf* mix, where both strains really merged and developed some new features that can be assumed as a mixed biofilm, while reaching the maturation phase (Figure 7C,D and Figure 8). However, further investigations are needed to assure that the reduction of surface area in both strains is only related with the merged shape that do not allow to distinguish the distinct morphology of the two colonies. Specifically, the *Pseudomonas* strain belonging to the *fluorescens* complex is considered the most promising group of PGPR involved in the biocontrol of plant diseases for its antifungal metabolites production [42], despite this trait is almost absent in the SERP1 strain used for the present co-growth test. In addition, certain *P. fluorescens* strains can detoxify organic and inorganic pollutants, combating heavy metal pollution and bioremediating pesticides [56,57]. Regarding *Penicillium ochrochloron* here studied, the Ni tolerance and the metal uptake ability were already documented [24,40]. Moreover, *P. ochrochloron* is a metal-resistant fungus [58], highly tolerant to copper and other metals [59].

Although the analysis of bacteria and fungi allowed defining their individual properties, and potential implications and applications in bioremediation as previously described, the development of integrated protocols, jointly exploiting fungi and bacteria, requires and adequate knowledge of their mutual behavior in the microbial consortium. To date, few works investigated the possible BFIs and synergy between rhizosphere PGP bacteria and microfungi [60,61].

The monitoring of the fungal and bacterial abundance in the in vivo experimentation evidenced how the soils went through an intermediate stage before stabilization. After one month, in fact, the presence of typical environmental fungal strains such as *Neurospora* sp., *Mucor* sp., *Trichoderma* sp. and *Aspergillus* sp. was reported, and it was particularly high in the Control tests where no fungi were inoculated. Similarly, we can affirm that bacterial diversity traced that observed in other serpentinitic soils [24]. The high abundance of the genus *Pseudomonas* sp. is affected by the inoculum of 10^8^ CFU mL^−1^.

However, twelve months after inoculation (and six months after transplanting) data on fungi were more homogeneous. Fungi, in fact, were equally distributed in each test group, *Penicillium* cfr. *ochrochloron* was the most abundant strain in each treatment and some morphotypes disappeared (*Aspergillus* sp. and *Neurospora* sp.). This is probably due to the fungal strains’ competition, which favored the inoculated strain over time, allowing its aerial dispersion and colonization of sterile near soils of control tests.

On the contrary, the bacterial community showed a greater dominance of a few genera: *Erwinia* sp. in all treatments (deriving from original soil microbial strains), *Pseudomonas* sp. (BI and MIX), and *Bacillus* sp. (FI and MIX). In this case, the competition between microorganisms seemed to favor a ubiquitous strain such as *Erwinia* sp. isolated on both serpentinite and non-serpentinite sites, in rhizospheric and adjacent bare soil [24]. Previously, PGP and Ni tolerance tests performed on bacterial strains isolated from serpentinite soil [24] belonging to *Erwinia* sp. showed significant PGPR traits (production of siderophores and solubilization of phosphates). *Erwinia* tolerated up to 5 mM of Ni compared to *Pseudomonas* sp. and *Streptomyces* sp. (up to 15 mM), although it was more competitive from an ecological point of view.

Plants seemed to have a good response to bacterial and fungal inoculum after six months from transplanting in pots. However, in controls tests, where *Erwinia* genus is dominant, plant growth appeared modest compared to the other treatments. This might be related with the low ability of this microbial strain to alleviate root stress linked to high metal concentrations. Further investigations are required to clarify the interaction between *Erwinia* and roots and among other fungal strains in co-growth to clarify its possible synergistic traits. Potential *Erwinia* ‘rhizosphere effect’, described for other microbial strains where root system could promote the bacterial and fungal proliferation [62,63,64,65,66] should be investigated.

## 5. Conclusions

In conclusion, we identified the PGP strains of *P. ochrochloron* and *P. fluorescens* isolated from the rhizosphere of *A. utriculata* as able to synergically co-grow in a mixed culture developing a biofilm where the two microorganisms merge, reaching the mature stage. Hence, these strains have been employed in the in vivo experimentation on metalliferous soil to directly evaluate their potential synergic role in the rhizosphere of *A. utriculata* for phytoremediation purposes. Inocula of MIX and *Penicillium ochrochloron* show the positive effect.

This synergistic fungi-bacteria co-growth opens new perspectives in plants’ growth promotion and soil bioremediation at the rhizosphere level. The achieved results suggest the possible use of mixed inocula to improve the metal uptake of the associated hyperaccumulator plant species in metal-contaminated soils. Moreover, our study evaluated and underlined the relevance and effectiveness of the agar overlay, the agar plugs, and the distance co-growth screening tests for selecting microorganism consortia for biotechnological purposes.

## Figures and Tables

**Table 1:** Chemical composition of the studied soils (Avg = average; M = median; Min = minimum; Max = maximum). Major and minor elements are reported as oxide wt %; selected PTEs are reported in mg/kg [46].

Type	Data	MgO	Al_2_O_3_	SiO_2_	CaO	TiO_2_	MnOt	FeOt	V	Cr	Co	Ni	Cu	Zn
A horizon	Avg	20.84	7.71	53.04	1.1	0.76	0.19	9.87	80	1290	110	710	20	150
	M	20.71	7.92	52.97	1.09	0.77	019	9.58	100	1460	110	860	20	80
	Min	15.9	6.24	51.14	0.97	0.69	0.16	9.53	10	620	30	270	10	70
	Max	26.03	8.75	55.07	1.41	0.83	0.22	10.79	140	1690	150	1050	30	430
C horizon	Avg	21.41	7.14	50.82	0.87	0.7	0.22	9.88	90	1840	190	1180	30	150
	M	21.41	6.99	49.82	0.88	7.71	0.22	9.85	100	2350	250	1140	20	70
	Min	14.3	6.79	48.23	0.46	0.32	0.22	9.27	30	660	30	290	10	50
	Max	28.52	7.79	55.42	1.27	1.04	0.22	10.54	130	2510	290	1890	80	480

**Table 2:** Estimation of the surface area of bacterial and fungal colonies in control plates and in different distance growth treatments. Data are mean ± SD. N = 3 each colony. *Po*: *P. ochrochloron*; *Th*: *T. harzianum*; *Pf*: *P. fluorescens*; *Sv*: *S. vinaceus.*

Colony Area (mm^2^)				
Mixes	*Po*	*Th*	*Pf*	*Sv*
Control	4372.39 ± 432.76	6361.73 ± 0.00	1587.07 ± 444.04	1669.45 ± 447.18
*Po-Pf* mix	2818.81 ± 514.19	-	713.65 ± 107.22	-
*Po*-*Sv* mix	2478.35 ± 236.31	-	-	1536.92 ± 655.43
*Th*-*Pf* mix	-	4042.99 ± 1049.64	377.69 ± 152.39	-
*Th-Sv* mix	-	4284.50 ± 216.29	-	768.90 ± 160.57

**Table 3:** Mutual inhibition of bacterial and fungal growth at different distance growth treatments. N = 3 each colony. *Po*: *P. ochrochloron*; *Th*: *T. harzianum*; *Pf*: *P. fluorescens*; *Sv*: *S. vinaceus.*

Percentage of Inhibition (%)								
	*Po-Pf* Mix		*Po-Sv* Mix		*Th-Pf* Mix		*Th-Sv* Mix	
Parameter	*Po*	*Pf*	*Po*	*Sv*	*Th*	*Pf*	*Th*	*Sv*
Area (mm^2^)	35.53	55.03	43.32	7.94	36.45	76.20	32.65	53.94

## Data Availability

Not applicable.
